# Tunneling nanotubes mediate rescue of prematurely senescent endothelial cells by endothelial progenitors: exchange of lysosomal pool

**DOI:** 10.18632/aging.100341

**Published:** 2011-06-23

**Authors:** Kaoru Yasuda, Anupama Khandare, Leonid Burianovskyy, Shoichi Maruyama, Frank Zhang, Alberto Nasjletti, Michael S Goligorsky

**Affiliations:** ^1^ Departments of Medicine, Pharmacology and Physiology, Renal Research Institute, New York Medical College, Valhalla, New York, USA.; ^2^ Department of Nephrology, Nagoya University Graduate School of Medicine, Nagoya, Japan

**Keywords:** lysosomes, endothelial progenitor cells, vasculopathy, stress-induced premature cell senescence

## Abstract

Although therapeutic effect of adoptive transfer of endothelial progenitor cells (EPC) has been well-substantiated, the actual engraftment is relatively low compared to a robust functional improvement of vasculopathy. Cellular mechanisms governing this action remain elusive. A recently discovered cell-cell communication via tunneling nanotube (TNT) formation is capable of transferring mitochondria and lysosomes between the cells – “organellar diakinesis”. Based on the previous demonstration of lysosomal dysfunction in endothelial cells exposed to AGE-modified collagen I, we inquired whether TNT mechanism may be involved in EPC-mediated repair of stressed endothelial cells. Here we demonstrate that EPC selectively and multiplicatively establish TNT communication with stressed endothelia. The guidance cues for the selectivity are provided by exofacially exposed phosphatidylserine moieties. Lysosomal transfer is associated with the preservation of lysosomal pH gradient, functionally reconstituting lysosomal pool of stressed cells and improving endothelial cell viability, reducing premature senescence and apoptosis. In vivo, adoptive transfer of EPC to streptozotocin-diabetic mice results in a TNT-dependent reduction of senescent endothelial cells and correction of endothelium-dependent vasorelaxation. Collectively, these data establish a selective multiplicative effect of TNT between EPC and stressed endothelia, reconstitution of the lysosomal pool, and improved viability and function of stressed endothelia.

## INTRODUCTION

Diseases as diverse as diabetes, atherosclerosis, chronic kidney disease and multitude of other conditions associated with oxidative stress result in stress-induced premature senescence (SIPS) of vascular endothelium, which contributes to development of vasculopathy [1-3]. We have recently demonstrated that the diabetic milieu triggers collapse of lysosomal pH gradient, lysosomal permeabilization, and frustrates autophagy – all pathogenetically linked to developing stress-induced premature senescence of vascular endothelium and vasculopathy [4, 5]. The role of autophagy in delaying cellular and organismal aging has been extensively studied [rev in: 6] Furthermore, adoptive transfer of bone marrow-derived stem cells markedly improved macro- and microvasculopathy [7].

The role of EPC in vascular repair has been well-substantiated [rev in: 8]. One issue that confronts multitude of investigations in this field is the scarcity of engraftment by transplanted EPC, usually averaging 1-2% [9-12]. Similarly, in mesenchymal stem cell transplantation – low engraftment associated with measurable functional effect was also noted – less than 1% in osteogeneis imperfecta (OI) trial [13]. This discrepancy explains an intense search for indirect mechanism(s) of vascular repair by EPC that could reconcile the scarcity of engrafted cells with the notable functional response. How this could be accomplished represented the goal of the present investigation.

Tunneling nanotubes (TNT) formation between cultured cells has been described [14] and proved to be a viable mechanism of organellar exchange between the partners. This mechanism has been shown to account for mitochondrial transfer between adult stem cells and somatic cells and rescue their respiration [15]. This mechanism is believed to play a significant role in intercellular communication, although it remains technically difficult to morphologically document it in vivo [16].

In our previous studies [17] human umbilical vein endothelial cells (HUVEC) were co-cultured with EPC, each cell-type labeled with differentially emitting fluorophores, and it was observed that exchange of fluorophores occurs under basal conditions. EPC-to-HUVEC exchange, however, increased 3-fold after exposure of HUVEC to the non-lethal cytotoxic concentration of adriamycin. TNT exchange mechanism was associated with the transport of mitochondria to HUVEC and improvement of their survival. Here, we analyzed the potential involvement of TNT mechanism in improved viability and amelioration of stress-induced premature senescence of endothelial cells exposed to a diabetic-like milieu through the ability of intact EPC to transfer lysosomes to HUVEC (and vice versa), examined the transfer ratio and *in vitro* and *in vivo* functional consequences of this transfer.

## RESULTS

### Effects of AGE-modified collagen I on the viability of HUVEC

**Figure 1:**
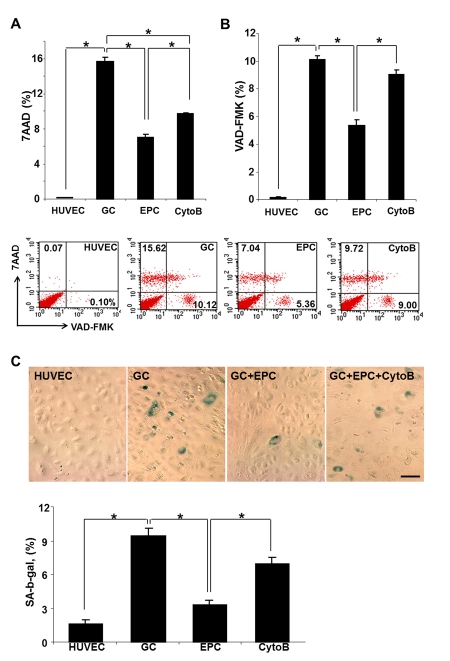
Cumulative death (A), apoptosis (B) and premature senescence (C) of stressed endothelial cells and effects of co-culture with EPC. (**A** and **B**) Representative flow cytometric analysis of VAD-FMK positive cells and 7AAD positive cells. Percentage positive cells are shown. (**C**) Representative images of cells expressing senescence-associated β-galactosidase. Note that co-culture with EPC improved viability and reduced the proportion of senescent endothelial cells, whereas pre-treatment of EPC with cytochalasin B (CytoB) partially annihilated this effect. Asterisks depict p< 0.05 (**A**) and (**B**) n=4; (**C**) n=9, Bars 30μm.

In the previous studies, we have demonstrated that non-enzymatically glycated long-lived protein exemplified by the collagen I (GC) resulted in lysosomal dysfunction, reduced viability of HUVEC and their premature senescence [4, 5, 18]. This observation was confirmed and expanded to examine the effect of co-cultured intact EPC on these parameters. In studies utilizing FACS analysis for detection of non-viable cells (7-AAD and VAD-FMK), application of GC for up to 3 days resulted in a dramatic increase in the population of non-viable and apoptotic cells (Fig. 1, A and B), whereas studies detecting SA-β-galactosidase expressing cells confirmed the increase in the population of prematurely senescent endothelial cells. The proportion of dead and prematurely senescent cells was decreased approximately 2-3-fold, respectively, after 24 h co-incubation of HUVEC with intact EPC. These data suggested that co-incubation with EPC was sufficient to improve survival and functionality of stressed endothelial cells.

We next examined the necessity of TNT formation as a potential rescue mechanism. Since the formation of TNT occurs in part via actin polymerization and filopodial elongation, and cytochalasin B at nanomolar concentrations has been shown to disrupt TNT formation and exchange without affecting endocytosis [19], we utilized this approach to pretreat EPC used in co-culture experiments prior to co-incubation with HUVEC affected by GC. Cytochalasin B (350 nM) almost completely negated the effects of EPC on the viability of HUVEC exposed to GC (Fig. 1, A - C). These findings suggested that EPC can partially rescue HUVEC after GC-induced stress and that the mode of EPC action could be related to the nanomolar cytochalasin B-inhibitable formation of TNT.

### TNT formation between HUVEC and EPC

**Figure 2:**
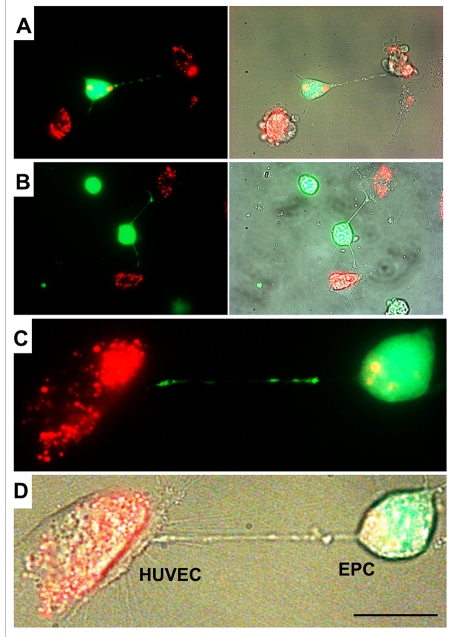
A gallery of images of TNT formation between HUVEC and EPC. (**A**, **B**, **C** and **D**) Images depict scarce transfer of lysotracker red-labeled lysosomes from stressed HUVEC to EPC labeled with CFDA SE green. Note that multiple TNT exist between two cell types, only a few of them convey lysosomes. Panels **C** and **D** depict enlarged fluorescence and bright-field images of HUVEC and EPC. Bars 20μm.

**Figure 3:**
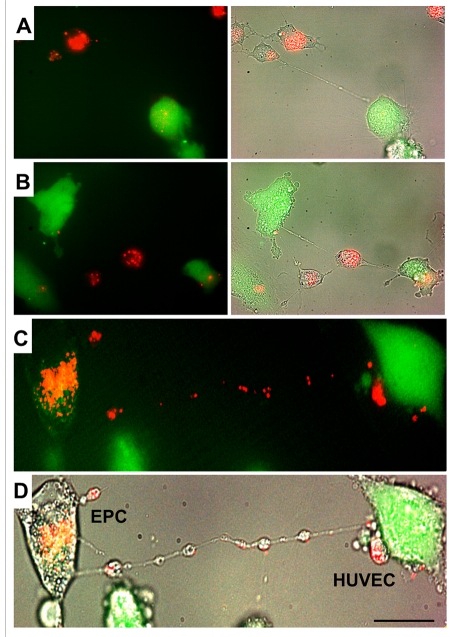
A gallery of images of TNT formation between HUVEC and EPC. (**A**, **B**, **C and D**) Typical examples of a robust transfer of lysosomes (lysotracker red) from EPC to stressed HUVEC labeled with CFDA SE green. Panels **C** and **D** depict fluorescence and bright-field images of HUVEC and EPC. Bars 20μm.

**Figure 4:**
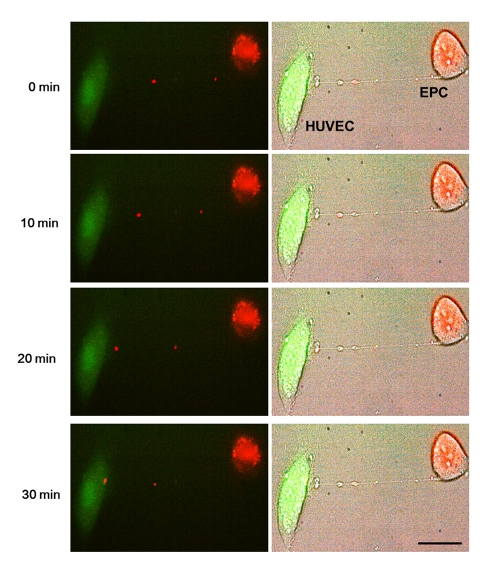
Representative time-lapse sequence of images illustrating a rapid transfer of lysosomes from an EPC to a stressed HUVEC. Time-lapse images of transfer of red-labeled lysosomes from EPC to stressed HUVEC labeled with CFDA SE green. Images were taken every 5 minutes. Left panels are fluorescence images and right panels are corresponding bright-field images.Bars 20μm.

Differential labeling of HUVEC and EPC with the Celltracker CFDA and Lysotracker were used to obtain direct evidence of TNT formation between these cells and lysosomal TNT exchange. Figures 2 and 3 provide respective galleries of typical images of bidirectional exchange between EPC and HUVEC. As shown in Fig 2, there was TNT-mediated lysosomal exchange in the direction HUVEC-to-EPC, however, the opposite direction of exchange was much more robust (Fig. 3), as was further quantitatively confirmed using FACS analysis. Figure 4 illustrates the time-lapse sequence of images demonstrating a rapid transfer of lysosomes from EPC to stressed HUVEC with an average rate of 1um/min.

### FACS analysis of lysosomal exchange between EPC and HUVEC: the basis for TNT selectivity of exchange

**Figure 5:**
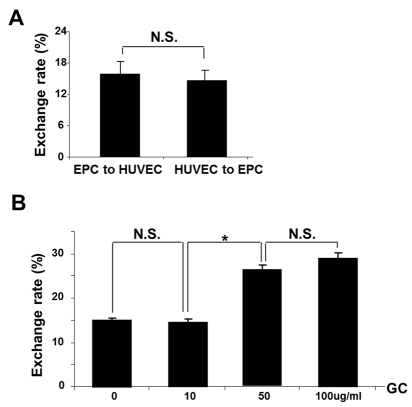
FACS analysis of bidirectional TNT lysosomal transfer between EPC and control (A) or stressed (B) HUVEC (n=5). HUVEC were cultured on 60mm dishes and GC was added at each concentration for 72 h of co-culture with EPC. Native collagen I was added in control (0ug/ml) group instead of GC. Note that exposure to 50 ug/ml GC and above resulted in the increased exchange rate at 24 h of co-culture. Asterisks depict p< 0.05; n=4. NS, not significant.

**Figure 6:**
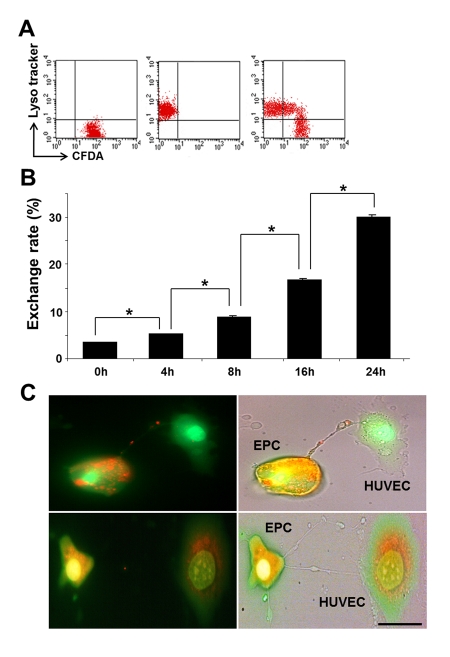
Time course of TNT exchange of lysosomes between co-cultured EPC and GC-stressed HUVEC and preservation of lysosomal pH gradient in the process of exchange. (**A**) A typical FACS analysis distribution of Celltracker and Lysotracker in the cells. Double-labeled cells indicated successful transfer. (**B**) Time-course of lysosomal transfer from EPC to stressed HUVEC. An increase in exchange rate occurred with time of co-culture. Asterisks depict p< 0.05; n=4. (**C**) Acridine orange labeling the low-pH compartment in EPC (orange), but lysosmal pH in stressed HUVEC is collapsed (green, upper panel). pH gradient persisted during the transfer of lysosomes to stressed HUVEC (upper panel). The lower panel illustrates the conclusion steps of lysosomal transfer with the restoration of lysosomal pH gradient in stressed HUVEC. Bars 20 μm.

FACS analyses demonstrated basal level of exchange between intact EPC and HUVEC (Fig. 5, A) that occurred with equal frequency. In contrast, the TNT formation occurred at a higher frequency and preferentially between intact and injured cells. The time-course and exchange rate between intact-to-stressed cells is summarized in Fig. 5, B and Fig. 6,B. The process resulted in a 1:3 exchange between EPC and HUVEC after 24h co-culture and showed that injurious levels of GC (concentrations of 50-100 ug/ml) [18] increased the selectivity of exchange.

The minimal requirement for the lysosomal transfer to have an impact on a recipient cell consists in the ability to preserve the integrity of the lysosomal membrane during the transit through a TNT. Egress of highly acidic lysosomes from EPC along the TNT toward GC-treated HUVEC, which express lysosomes with the collapsed pH gradient (4), was examined using acridine orange, which labels acidic compartments in EPC (red). As illustrated in Fig. 6,C, lysosomal pH was preserved in the process of TNT transfer from EPC to stressed HUVEC (upper panel) and the lysosomal pool of HUVEC showed acquisition of the previously collapsed low-pH lysosomal compartment (lower panel).

**Figure 7:**
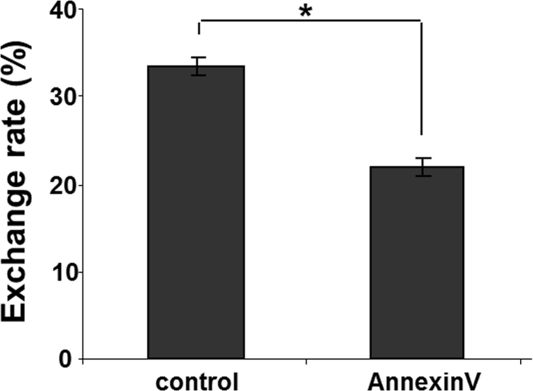
Shielding of exofacial phosphatidylserine domains by pretreatment of stressed HUVEC with annexin A5 reduces the rate of lysosomal exchange from intact EPC. Flow cytometric quantification was performed. Control – the rate of exchange in stressed HUVEC. Annexin A5 – same as control after pretreatment with Annexin A5 (BD Bioscience, San Diego, CA). Asterisks depict p< 0.05;n=6.

This finding prompted a search for a potential mechanism(s) of the selectivity of TNT formation between intact and stressed cells. We hypothesized that one the following mechanisms could be involved in guiding TNT formation: a) attraction via Weibel-Palade body-released products by stressed endothelial cells; and b) via the cell surface-exposed phosphatidylserine (PS) moieties. Analysis of the first hypothetical mechanism using an inhibitor of Weibel-Palade bodies exocytosis (20) did not yield a clear-cut support for their involvement in selective TNT formation (data not shown). In contrast, shielding of the exposed PS with annexin A5 (1:100 dilution, Annexin V Alexa Fluor 594 conjugate, Molecular Probes, Eugene, OR) resulted in the loss of a gradient and significant equalization of TNT formation and exchange (Fig. 7).

### Formation of autolysosomes and the effect of TNT exchange on this process

**Figure 8:**
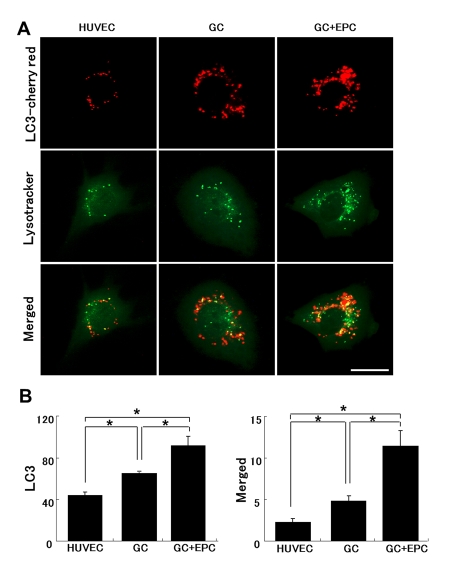
Lysosomal and autophagosomal punctae in control or stressed (GC) HUVEC. (**A**) Representative images of HUVEC expressing LC3-cherry red (autophagosomal marker, upper raw) and lysotracker (middle raw) under basal conditions, following application of GC in the absence or presence of intact EPC. The lower panel depicts corresponding merged images. Co-localization of autophagosomal and lysosomal markers indicates their fusion (autolysosomal formation), which has been shown to be subverted in stressed HUVEC (Bars 20μm). (**B**) Quantitative summary of autophagosomal punctae and their fusion with lysosomes (co-localized). Image J software (National Institutes of Health) was used for analysis. Asterisks p< 0.05; n=5.

Based on the previous finding of subverted autophagy in GC-stressed HUVEC, we next inquired whether TNT-mediated lysosomal exchange may restore this function. HUVEC transfected with LC3-cherry red fluorescent protein were exposed to GC and the density of autophagosomes, lysosomes (labeled with Lysotracker green), and autolysosomes (double-staining) was quantified (Fig. 8). Co-culture with EPC increased the pool of autophagosomes and autolysosomes in stressed HUVEC. The data are consistent with improved autophagy in stressed HUVEC in co-culture with EPC.

### In vivo effects of adoptively transferred EPC: role of TNT formation

**Figure 9:**
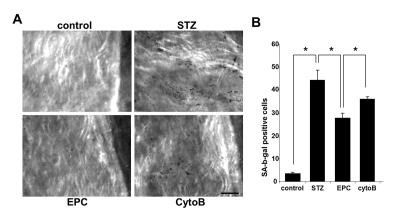
Senescence-associated β-galactosidase stained endothelial cells in *en face* aortic preparations obtained from control and STZ mice, non-treated or treated with intact EPC or with EPC preincubated with cytochalasin B (CytoB). (**A**) Representative images obtained using intravital microscopy. (Nikon, Melville, NY) Bars 50μm. (**B**)Quantitative analysis of senescence-associated β-galactosidase-stained endothelial cells. Asterisks indicate p< 0.05; n=5.

**Figure 10:**
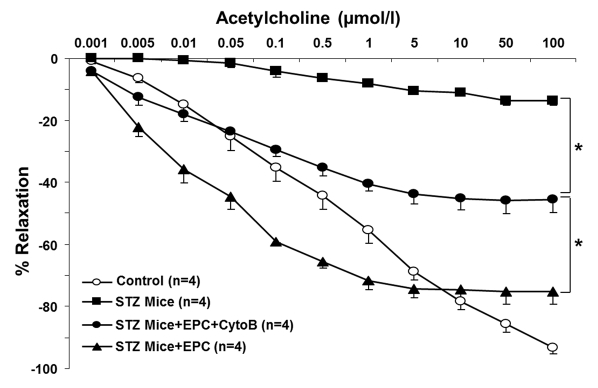
Acetylcholine-induced relaxation of aortic rings obtained from control and STZ mice, non-treated and treated with intact EPC or with EPC preincubated with cytochalasin B (CytoB). Cumulative dose-response curves of acetylcholine-induced vasorelaxation in phenylephrine preconstricted aortic rings. Significant improvement in acetylcholine-induced vasorelaxation was documented for intact EPC, followed by CytoB preincubated EPC and STZ alone. Asterisks p< 0.05

To gain insights into an in vivo efficacy of TNT transfer in salvaging endothelial cells exposed to a diabetic milieu, the effect of EPC was examined in a streptozotocin-induced diabetes mouse model. Previous studies using this model have demonstrated that MSC improved several complications of diabetes [21]. Here, we examined the acetylcholine-induced relaxation of vascular rings and the density of SA-β-gal-positive cells in *en face* aortic preparations obtained from EPC-treated and non-treated animals. In an additional series of experiments, adoptive transfer of EPC was preceded by their pretreatment with cytochalasin B at 350 nM. The number of SA-β-gal-positive endothelial cells in *en face* aortic preparations obtained from STZ mice was dramatically increased, whereas adoptive transfer of EPC curtailed it (Fig. 9). The observed effect of EPC was significantly less pronounced in mice receiving cytochalasin B-pretreated EPC. In accord with these findings, acetylcholine-induced relaxation of aortic rings was significantly improved in recipients of adoptively transferred EPC, whereas EPC pretreatment with cytochalasin B diminished this endothelium-dependent relaxation (Fig. 10).

## DISCUSSION

The data presented herein demonstrated a) the selectivity of TNT formation, which is annexin A5-dependent, b) the ability to transfer intact lysosomes from EPC to endothelial cells exposed to a diabetes-like milieu without the loss of their integrity, which resulted in improved lysosomal-autophagosomal fusion and led to c) salvage of endothelial cells from premature senescence and apoptosis, and provided d) indirect *in vivo* support of the role played by TNT formation between EPC and stressed vascular endothelium in improving endothelial dysfunction. These findings raise several important issues.

TNT-mediated cell-to-cell transfer of organelles, lysosomes and mitochondria, which we propose to refer to as “organellar diakinesis”, represents one of the several modes of information exchange between cells; others are exemplified by cell-cell fusion and microvesicular exchange [22]. One of the distinguishing features of TNT-mediated exchange is its selectivity toward damaged cells and the multiplicative effect – repeated time-dependent rounds of exchange between EPC and approximately 3 endothelial cells after 24h in co-culture. Although the intricate mechanisms for the selectivity toward stressed cells require further detailed exploration, the data support the role of the externalized PS domains in guiding TNT destination – when these domains are shielded by the presence of annexin A5 [23], selectivity of exchange is inhibited. It is possible to envisage a scenario whereby the baseline rate of TNT formation between intact cells is guided by a stochastic flip-flop of PS, and the process gains in intensity with the increasing frequency of these events in the stressed or injured cells. This scenario may bare similarities with macrophage phagocytosis of apoptotic cells, the process that has been traced to macrophage CD36 scavenger receptor engaging PS or oxidized PS and, to a lesser extent, oxidized phosphatidylcholine, expressed on the exofacial surface of the plasma membrane [24, 25]. Such a scenario would be consistent with a model depicting TNT formation as an example of frustrated phagocytosis [26]. In this context it is noteworthy that Annexin A5 binds with a near-equal affinity to PS and oxidized PS [25]. In addition, recognition of the exofacially expressed PS, the well-known “eat-me” signal, may occur via other clearance receptors like LOX-1, SRB1, SRA, CD68, CD14 or members of type I membrane proteins, T cell immunoglobulin and mucin-domain-containing molecules (Tim-1, also known as kidney injury molecule, and Tim-4), and stabilin-2, a hyaluronic acid receptor for endocytosis [27-29]. All these possibilities, together with other “find-me” and “eat-me” signals, will require further scrutiny.

Regardless of the potentially multifaceted mechanisms of TNT guidance, the data demonstrated a distinct improvement of HUVEC function in cell co-culture experiments and in vivo after adoptive transfer of EPC. Considering the fact that, on the one hand, expression of PS on the outer leaflet of the plasma membrane is one of the hallmarks of cells committed to apoptosis and, on the other, represents a guidance cue for targeted TNT formation, the observed functional improvement raises the question of potential reversibility of apoptotic commitment. Indeed, when TNT formation was inhibited by an F-actin-depolymerizing agent, nanomolar cytochalasin B, stressed HUVEC viability in co-culture with intact EPC was significantly reduced, the number of prematurely senescent endothelial cells in *en face* aortae was not diminished, as it occurred with adoptive transfer of intact EPC, and acetylcholine-induced vasorelaxation showed a significantly lesser improvement. The data would argue that the commitment to apoptosis, as judged by the appearance of exofacial PS, is not unconditionally finite and can be disrupted by TNT-mediated organellar diakinesis. In fact, the role of lysosomal dysfunction in facilitating apoptosis has been long-recognized [30-32]. Lysosomal dysfunction and cell death can be attenuated by serine protease inhibitor 2A [33], heat shock protein 70 [34], or as shown in B-lymphocytes, by contact with follicular dendritic cells [35]. The finding of TNT-mediated reconstitution of the lysosomal compartment in stressed endothelial cells associated with improved viability represents, to the best of our knowledge, the first demonstration of the therapeutic capacity of this mechanism under pathological conditions.

It is conceivable that TNT exchange of mitochondrial pool takes place in parallel with the lysosomal exchange described herein. Mitochondrial compartment during TNT exchange between stem and recipient somatic cells in diabetic mice has been improved through exchange of these organelles [15]. On the other hand, the contribution of impaired autophagy, as would occur with lysosomal dysfunction, to abnormalities in mitochondrial pool has been convincingly demonstrated in 2 genetically engineered animal models of auto-phagosomal dysgenesis [36]. Therefore, the focus of present studies was on the lysosomal compartment.

Two types of TNT have been encountered in human macrophages: <0.7 um TNT supporting unidirectional transfer of plasma membrane constituents and thicker, >0.7 um TNT capable of transferring intracellular organelles [37]. In our co-culture EPC/HUVEC system, both types of TNT appear to be present. This would suggest that additional modes of EPC action could exist, including transport of plasma membrane proteins and/or lateral transfer of phospholipid components of the plasma membrane. These questions remain unexplored by the present studies.

Finally, data presented in this communication should raise the level of awareness as related to TNT formation and TNT-mediated exchange, and effects of these cellular communications on tissue regeneration should be thoroughly examined pharmacologically to reveal therapeutics that may facilitate TNT formation and information exchange, on the one hand, and, on the other, limit the use of such therapeutics that may hamper these processes and interfere with regeneration. A broad-based search for both types of compounds will represent the future challenge.

## MATERIALS AND METHODS

### Cell cultures

Human umbilical vein endothelial cells (HUVEC) were purchased from Clonetics (Walkersville, MD) and used between passages 3-7. Cells were cultured in EBM-2 medium (Cambrex Bio Science Walkersville, Inc., MD, USA) with EGM-2 SingleQuots supplements (Cambrex Bio Science Walkersville, Inc., MD, USA) and maintained at 37°C incubator with 5% CO_2_. Embryonic mouse EPC were obtained from Dr. A Hatzopoulos, Vanderbilt University [38] and cultured on 0.1% gelatin-coated plates in DMEM culture medium containing 20% heat-inactivated serum (55°C 30 minutes; Invitrogen, Carlsbad, CA), 0.1 mM 2-mercaptoethanol, 1 mM MEM nonessential amino acids (Invitrogen), 100 u/ml penicillin and 100 ug/ml streptomycin, 2 mM L-glutamine (Invitrogen) and 2 mM Hepes at pH 7.5. Cells were grown in tissue culture incubators at 37°C and 5% CO2. For selective inhibition of TNT formation, EPC were pretreated with cytochalasin B (350nM).

### Fluorescence microscopy and time-lapse microscopy of TNT formation

HUVEC used between passages 3-6 were cultured to about 75% subconfluence on gelatin-coated glass-bottom dishes (MatTek, Ashland, MA) and exposed to 50ug/ml glycated collagen I (GC) for 3 consecutive days, as previously detailed [17]. Prior to experiments cells were labeled with green fluorescent CFDA SE (*Invitrogen*, Eugene, OR). Cells were washed with fresh culture medium and co-cultured with EPC for the next 4-24 h. EPC were labeled with red fluorescent Lysotracker (Molecular Probes, Carlsbad, CA). Cells were washed, and presented to HUVEC cultures at concentration of one-tenth of HUVEC. In companion experiments, the labeling with fluorophores was reversed: HUVEC were labeled with Lysotracker and EPC with CFDA SE. Images were obtained using a compound Nikon microscope (TE-2000U microscope equipped with equipped with a Spot Insight digital camera (Diagnostic Instruments). For time-lapse videomicroscopy, images were acquired every 5 minutes. Monitoring lysosomal pH during TNT exchange was performed using a lysosomotropic weak base metachromatic fluorescent indicator acridine orange [30], as previously detailed [4].

### FACS analysis of lysosomal exchange

Exchange rate of Lysotracker was analyzed by FACS. CFDA labeled HUVEC treated with GC and Lysotracker-labeled EPC at the one-tenth of concentration of HUVEC were co-cultured on 60 mm dishes (Becton Dickinson, Franklin Lakes, NJ), and examined after 24hrs. Data were acquired using a FACScan cytometer equipped with a 488nm argon laser and a 635nm red diode laser and analyzed using CellQuest software (Becton Dickinson, Franklin Lakes, NJ). The set-up of FACScan was performed using unstained cells and HUVEC or EPC alone.

### Analysis of cell viability and senescence

To detect apoptotic and necrotic cells, FACS analysis using fluorescein isothiocyanate-Val-Ala-Asp (OMe)-fluoromethylketone (FITC-VAD-FMK, Calbiochem, La Jolla, CA) and 7-aminoactinomycin D (7-AAD, Invitrogen), respectively, was performed. Detection of cell senescence was accomplished by staining for senescence-associated beta-galactosidase (SA-β-gal), according to the previously published protocol [39].

### Formation of autophagosomes and autolysosomes

To observe the density of lysosomes and autophagosomes, as well as frequency of fusion to autolysosomes, HUVEC were analyzed by FACS or by fluorescence microscopy. Autophagosomes were detected by transfecting cells with light-chain 3 (LC3)-cherry fluorescent protein plasmid [40], whereas lysosomes were stained with green Lysotracker according to manufacturer's instructions.

### In vivo adoptive transfer of EPC in streptozotocin (STZ)-induced diabetes in mice: role of TNT formation

All animal protocols were conducted in accord with the National Institutes of Health (NIH) guidance and were approved by the Institutional Animal Care and Use Committee.Diabetes mellitus (DM) was induced in 8-week-old male FVB/NJ (Jackson Laboratory, Bar Harbor, ME) with intraperitoneal injection of STZ (Sigma Chemical Co, Saint Louis, MO) of 50mg/kg for 5 consecutive days. Successful induction of diabetes was monitored by testing glucose levels in plasma and urine. For adoptive transfer, EPC obtained from companion mice were cultured from bone marrow mononuclear cells. Briefly, bone marrow (BM) mononuclear cells were obtained by flushing the tibias and femurs of FVB/NJ mice with PBS followed by density gradient centrifugation in Histopaque-1077 (Sigma). BM mononuclear cells were cultured in EGM-2 media on dishes coated with 10ug/ml pronectin (Sigma, St Louis, MO). After 3 days in culture, non-adherent cells were removed, and medium exchanged every 2 days. Mice was treated with EPC by adoptive transfer of approximately 1x10^6^ cultured EPC injected into the circulation via the tail vein on days 30 and 40 post-STZ. An additional group of mice received equal number of EPC pretreated with cytochalasin B (350nM). Non-treated mice received an injection of normal saline. On the days 0, 30, 40, and 50, mice were placed in metabolic cages for 24-h urine collection. Mice were sacrificed at day 50 after STZ injection. On the day of sacrifice, after Ketamin/Xylazine anesthesia, blood was obtained through left ventricular puncture and animals were perfused with normal saline followed by perfusion-fixation with 4% paraformaldehyde for morphologic studies.

### Relaxation of aortic rings

The descending thoracic aorta from control and STZ-diabetic mice was isolated and cut into cylindrical segments which were mounted on a wire-myograph containing Krebs buffer gassed with 95% O_2_-5% CO_2_ for recording of isometric tension [41]. The vessels were preconstricted with phenylephrine to 70% of maximal response and used for measurements of acetylcholine (0.001-100 μmol/L)-induced vasorelaxation.

### Statistical Analysis

Statistical analysis was performed with GraphPad Prism 4.0 software (Software Inc, San Diego, CA). Statistical significance was determined by an unpaired, 2-tailed Student's *t* test or ANOVA, as appropriate. Post-hoc analysis for multiple group comparisons was performed using Bonferroni method. A P value of less than 0.05 was considered statistically significant. All data were expressed as mean ±SEM.
